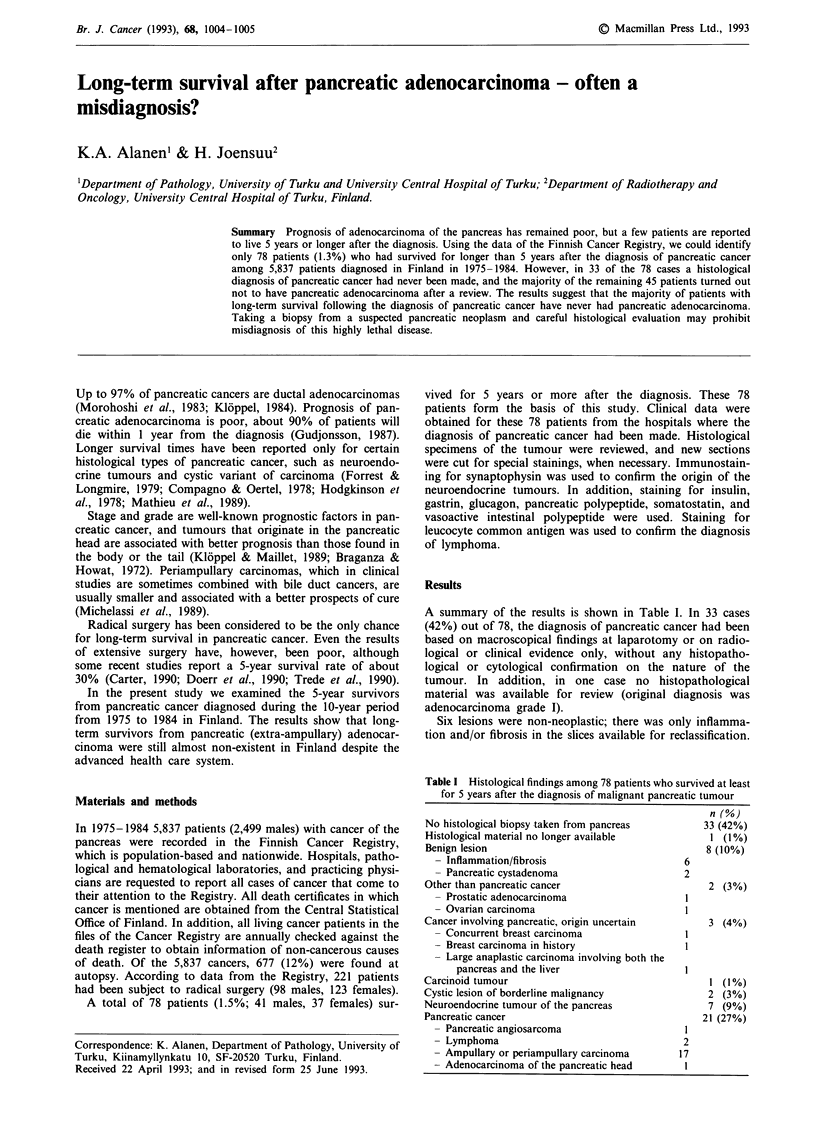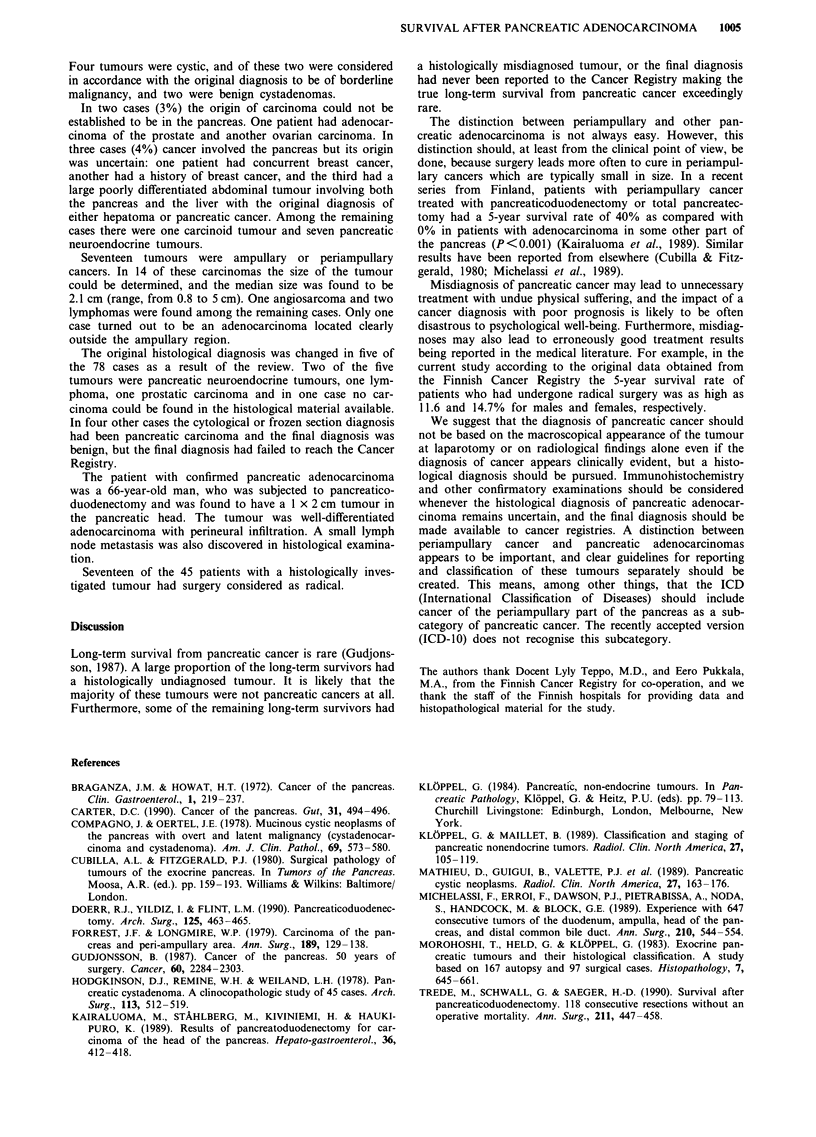# Long-term survival after pancreatic adenocarcinoma--often a misdiagnosis?

**DOI:** 10.1038/bjc.1993.469

**Published:** 1993-11

**Authors:** K. A. Alanen, H. Joensuu

**Affiliations:** Department of Pathology, University of Turku, Finland.

## Abstract

Prognosis of adenocarcinoma of the pancreas has remained poor, but a few patients are reported to live 5 years or longer after the diagnosis. Using the data of the Finnish Cancer Registry, we could identify only 78 patients (1.3%) who had survived for longer than 5 years after the diagnosis of pancreatic cancer among 5,837 patients diagnosed in Finland in 1975-1984. However, in 33 of the 78 cases a histological diagnosis of pancreatic cancer had never been made, and the majority of the remaining 45 patients turned out not to have pancreatic adenocarcinoma after a review. The results suggest that the majority of patients with long-term survival following the diagnosis of pancreatic cancer have never had pancreatic adenocarcinoma. Taking a biopsy from a suspected pancreatic neoplasm and careful histological evaluation may prohibit misdiagnosis of this highly lethal disease.


Br. J. Cancer (1993), 68, 1004-1005 © Macmillan Press Ltd., 1993

### Long-term survival after pancreatic adenocarcinoma – often a misdiagnosis?

K.A. Alanen[1] & H. Joensuu[2]

[1]*Department of Pathology, University of Turku and University Central Hospital of Turku;* [2]*Department of Radiotherapy and Oncology, University Central Hospital of Turku, Finland.*

**Summary** Prognosis of adenocarcinoma of the pancreas has remained poor, but a few patients are reported to live 5 years or longer after the diagnosis. Using the data of the Finnish Cancer Registry, we could identify only 78 patients (1.3%) who had survived for longer than 5 years after the diagnosis of pancreatic cancer among 5,837 patients diagnosed in Finland in 1975–1984. However, in 33 of the 78 cases a histological diagnosis of pancreatic cancer had never been made, and the majority of the remaining 45 patients turned out not to have pancreatic adenocarcinoma after a review. The results suggest that the majority of patients with long-term survival following the diagnosis of pancreatic cancer have never had pancreatic adenocarcinoma. Taking a biopsy from a suspected pancreatic neoplasm and careful histological evaluation may prohibit misdiagnosis of this highly lethal disease.

Up to 97% of pancreatic cancers are ductal adenocarcinomas (Morohoshi *et al.*, 1983; Klöppel, 1984). Prognosis of pancreatic adenocarcinoma is poor, about 90% of patients will die within 1 year from the diagnosis (Gudjonsson, 1987). Longer survival times have been reported only for certain histological types of pancreatic cancer, such as neuroendocrine tumours and cystic variant of carcinoma (Forrest & Longmire, 1979; Compagno & Oertel, 1978; Hodgkinson *et al.*, 1978; Mathieu *et al.*, 1989).

Stage and grade are well-known prognostic factors in pancreatic cancer, and tumours that originate in the pancreatic head are associated with better prognosis than those found in the body or the tail (Klöppel & Maillet, 1989; Braganza & Howat, 1972). Periampullary carcinomas, which in clinical studies are sometimes combined with bile duct cancers, are usually smaller and associated with a better prospects of cure (Michelassi *et al.*, 1989).

Radical surgery has been considered to be the only chance for long-term survival in pancreatic cancer. Even the results of extensive surgery have, however, been poor, although some recent studies report a 5-year survival rate of about 30% (Carter, 1990; Doerr *et al.*, 1990; Trede *et al.*, 1990).

In the present study we examined the 5-year survivors from pancreatic cancer diagnosed during the 10-year period from 1975 to 1984 in Finland. The results show that long-term survivors from pancreatic (extra-ampullary) adenocarcinoma were still almost non-existent in Finland despite the advanced health care system.

### Materials and methods

In 1975–1984 5,837 patients (2,499 males) with cancer of the pancreas were recorded in the Finnish Cancer Registry, which is population-based and nationwide. Hospitals, pathological and hematological laboratories, and practicing physicians are requested to report all cases of cancer that come to their attention to the Registry. All death certificates in which cancer is mentioned are obtained from the Central Statistical Office of Finland. In addition, all living cancer patients in the files of the Cancer Registry are annually checked against the death register to obtain information of non-cancerous causes of death. Of the 5,837 cancers, 677 (12%) were found at autopsy. According to data from the Registry, 221 patients had been subject to radical surgery (98 males, 123 females).

A total of 78 patients (1.5%; 41 males, 37 females) survived for 5 years or more after the diagnosis. These 78 patients form the basis of this study. Clinical data were obtained for these 78 patients from the hospitals where the diagnosis of pancreatic cancer had been made. Histological specimens of the tumour were reviewed, and new sections were cut for special stainings, when necessary. Immunostaining for synaptophysin was used to confirm the origin of the neuroendocrine tumours. In addition, staining for insulin, gastrin, glucagon, pancreatic polypeptide, somatostatin, and vasoactive intestinal polypeptide were used. Staining for leucocyte common antigen was used to confirm the diagnosis of lymphoma.

Correspondence: K. Alanen, Department of Pathology, University of Turku, Kiinamyllynkatu 10, SF-20520 Turku, Finland.
Received 22 April 1993; and in revised form 25 June 1993.

### Results

A summary of the results is shown in Table I. In 33 cases (42%) out of 78, the diagnosis of pancreatic cancer had been based on macroscopical findings at laparotomy or on radiological or clinical evidence only, without any histopathological or cytological confirmation on the nature of the tumour. In addition, in one case no histopathological material was available for review (original diagnosis was adenocarcinoma grade I).

Six lesions were non-neoplastic; there was only inflammation and/or fibrosis in the slices available for reclassification.

**Table I** Histological findings among 78 patients who survived at least for 5 years after the diagnosis of malignant pancreatic tumour

| | | *n (%)* |
|---|---|---|
| No histological biopsy taken from pancreas | | 33 (42%) |
| Histological material no longer available | | 1 (1%) |
| Benign lesion | | 8 (10%) |
| – Inflammation/fibrosis | 6 | |
| – Pancreatic cystadenoma | 2 | |
| Other than pancreatic cancer | | 2 (3%) |
| – Prostatic adenocarcinoma | 1 | |
| – Ovarian carcinoma | 1 | |
| Cancer involving pancreatic, origin uncertain | | 3 (4%) |
| – Concurrent breast carcinoma | 1 | |
| – Breast carcinoma in history | 1 | |
| – Large anaplastic carcinoma involving both the pancreas and the liver | 1 | |
| Carcinoid tumour | | 1 (1%) |
| Cystic lesion of borderline malignancy | | 2 (3%) |
| Neuroendocrine tumour of the pancreas | | 7 (9%) |
| Pancreatic cancer | | 21 (27%) |
| – Pancreatic angiosarcoma | 1 | |
| – Lymphoma | 2 | |
| – Ampullary or periampullary carcinoma | 17 | |
| – Adenocarcinoma of the pancreatic head | 1 | |

Four tumours were cystic, and of these two were considered in accordance with the original diagnosis to be of borderline malignancy, and two were benign cystadenomas.

In two cases (3%) the origin of carcinoma could not be established to be in the pancreas. One patient had adenocarcinoma of the prostate and another ovarian carcinoma. In three cases (4%) cancer involved the pancreas but its origin was uncertain: one patient had concurrent breast cancer, another had a history of breast cancer, and the third had a large poorly differentiated abdominal tumour involving both the pancreas and the liver with the original diagnosis of either hepatoma or pancreatic cancer. Among the remaining cases there were one carcinoid tumour and seven pancreatic neuroendocrine tumours.

Seventeen tumours were ampullary or periampullary cancers. In 14 of these carcinomas the size of the tumour could be determined, and the median size was found to be 2.1 cm (range, from 0.8 to 5 cm). One angiosarcoma and two lymphomas were found among the remaining cases. Only one case turned out to be an adenocarcinoma located clearly outside the ampullary region.

The original histological diagnosis was changed in five of the 78 cases as a result of the review. Two of the five tumours were pancreatic neuroendocrine tumours, one lymphoma, one prostatic carcinoma and in one case no carcinoma could be found in the histological material available. In four other cases the cytological or frozen section diagnosis had been pancreatic carcinoma and the final diagnosis was benign, but the final diagnosis had failed to reach the Cancer Registry.

The patient with confirmed pancreatic adenocarcinoma was a 66-year-old man, who was subjected to pancreaticoduodenectomy and was found to have a 1 × 2 cm tumour in the pancreatic head. The tumour was well-differentiated adenocarcinoma with perineural infiltration. A small lymph node metastasis was also discovered in histological examination.

Seventeen of the 45 patients with a histologically investigated tumour had surgery considered as radical.

### Discussion

Long-term survival from pancreatic cancer is rare (Gudjonsson, 1987). A large proportion of the long-term survivors had a histologically undiagnosed tumour. It is likely that the majority of these tumours were not pancreatic cancers at all. Furthermore, some of the remaining long-term survivors had a histologically misdiagnosed tumour, or the final diagnosis had never been reported to the Cancer Registry making the true long-term survival from pancreatic cancer exceedingly rare.

The distinction between periampullary and other pancreatic adenocarcinoma is not always easy. However, this distinction should, at least from the clinical point of view, be done, because surgery leads more often to cure in periampullary cancers which are typically small in size. In a recent series from Finland, patients with periampullary cancer treated with pancreaticoduodenectomy or total pancreatectomy had a 5-year survival rate of 40% as compared with 0% in patients with adenocarcinoma in some other part of the pancreas ($P < 0.001$) (Kairaluoma *et al.*, 1989). Similar results have been reported from elsewhere (Cubilla & Fitzgerald, 1980; Michelassi *et al.*, 1989).

Misdiagnosis of pancreatic cancer may lead to unnecessary treatment with undue physical suffering, and the impact of a cancer diagnosis with poor prognosis is likely to be often disastrous to psychological well-being. Furthermore, misdiagnoses may also lead to erroneously good treatment results being reported in the medical literature. For example, in the current study according to the original data obtained from the Finnish Cancer Registry the 5-year survival rate of patients who had undergone radical surgery was as high as 11.6 and 14.7% for males and females, respectively.

We suggest that the diagnosis of pancreatic cancer should not be based on the macroscopical appearance of the tumour at laparotomy or on radiological findings alone even if the diagnosis of cancer appears clinically evident, but a histological diagnosis should be pursued. Immunohistochemistry and other confirmatory examinations should be considered whenever the histological diagnosis of pancreatic adenocarcinoma remains uncertain, and the final diagnosis should be made available to cancer registries. A distinction between periampullary cancer and pancreatic adenocarcinomas appears to be important, and clear guidelines for reporting and classification of these tumours separately should be created. This means, among other things, that the ICD (International Classification of Diseases) should include cancer of the periampullary part of the pancreas as a subcategory of pancreatic cancer. The recently accepted version (ICD-10) does not recognise this subcategory.

The authors thank Docent Lyly Teppo, M.D., and Eero Pukkala, M.A., from the Finnish Cancer Registry for co-operation, and we thank the staff of the Finnish hospitals for providing data and histopathological material for the study.

### References

BRAGANZA, J.M. & HOWAT, H.T. (1972). Cancer of the pancreas. *Clin. Gastroenterol.*, **1**, 219–237.

CARTER, D.C. (1990). Cancer of the pancreas. *Gut*, **31**, 494–496.

COMPAGNO, J. & OERTEL, J.E. (1978). Mucinous cystic neoplasms of the pancreas with overt and latent malignancy (cystadenocarcinoma and cystadenoma). *Am. J. Clin. Pathol.*, **69**, 573–580.

CUBILLA, A.L. & FITZGERALD, P.J. (1980). Surgical pathology of tumours of the exocrine pancreas. In *Tumors of the Pancreas.* Moosa, A.R. (ed.). pp. 159–193. Williams & Wilkins: Baltimore/London.

DOERR, R.J., YILDIZ, I. & FLINT, L.M. (1990). Pancreaticoduodenectomy. *Arch. Surg.*, **125**, 463–465.

FORREST, J.F. & LONGMIRE, W.P. (1979). Carcinoma of the pancreas and peri-ampullary area. *Ann. Surg.*, **189**, 129–138.

GUDJONSSON, B. (1987). Cancer of the pancreas. 50 years of surgery. *Cancer*, **60**, 2284–2303.

HODGKINSON, D.J., REMINE, W.H. & WEILAND, L.H. (1978). Pancreatic cystadenoma. A clinocopathologic study of 45 cases. *Arch. Surg.*, **113**, 512–519.

KAIRALUOMA, M., STÅHLBERG, M., KIVINIEMI, H. & HAUKIPURO, K. (1989). Results of pancreatoduodenectomy for carcinoma of the head of the pancreas. *Hepato-gastroenterol.*, **36**, 412–418.

KLÖPPEL, G. (1984). Pancreatic, non-endocrine tumours. In *Pancreatic Pathology*, Klöppel, G. & Heitz, P.U. (eds). pp. 79–113. Churchill Livingstone: Edinburgh, London, Melbourne, New York.

KLÖPPEL, G. & MAILLET, B. (1989). Classification and staging of pancreatic nonendocrine tumors. *Radiol. Clin. North America*, **27**, 105–119.

MATHIEU, D., GUIGUI, B., VALETTE, P.J. *et al.* (1989). Pancreatic cystic neoplasms. *Radiol. Clin. North America*, **27**, 163–176.

MICHELASSI, F., ERROI, F., DAWSON, P.J., PIETRABISSA, A., NODA, S., HANDCOCK, M. & BLOCK, G.E. (1989). Experience with 647 consecutive tumors of the duodenum, ampulla, head of the pancreas, and distal common bile duct. *Ann. Surg.*, **210**, 544–554.

MOROHOSHI, T., HELD, G. & KLÖPPEL, G. (1983). Exocrine pancreatic tumours and their histological classification. A study based on 167 autopsy and 97 surgical cases. *Histopathology*, **7**, 645–661.

TREDE, M., SCHWALL, G. & SAEGER, H.-D. (1990). Survival after pancreaticoduodenectomy. 118 consecutive resections without an operative mortality. *Ann. Surg.*, **211**, 447–458.